# INTEREST GROUP SESSION—HOSPICE, PALLIATIVE AND END-OF-LIFE CARE: ADDRESSING ADVANCED ILLNESS CHALLENGES THROUGH SYNERGY, PLANNING, COLLABORATION, AND ADVOCACY

**DOI:** 10.1093/geroni/igz038.643

**Published:** 2019-11-08

**Authors:** Faith P Hopp, Frances R Nedjat-Haiem

**Affiliations:** 1 Wayne State University, Detroit, Michigan, United States; 2 San Diego State University, School of Social Work, San Diego, California, United States

## Abstract

Advanced illness occurs when health conditions become serious, general health and functioning declines, and treatments begin to lose their impact. Advanced care planning (ACP) is critical, but challenges such as communication barriers, system fragmentation, and a lack of formal and informal supports can negatively influence ACP and quality of life. How can diverse groups of persons with advanced illness, their family members and friends have greater synergy when planning for care in the face of serious illness? What are the best practices for planning and provision of a spectrum of needed services and support during the dying process and afterwards? We extend a conversation important for formal and informal network members to transform the culture of ACP, death, dying, and bereavement for diverse vulnerable populations. For this symposium, we will present information on ACP and care for LGBT populations and older Latinos, those living in long-term care settings, and persons receiving Adult Protective Services (APS). We will discuss strategies for synergy, collaboration, and advocacy among persons with advanced illness and their formal and informal care networks as a means of reducing health disparities and promoting self-determination in the midst of advanced illness challenges.

